# Orbital invasion by an antrochoanal polyp in a factor V-deficient patient: A case report of diagnostic and surgical challenges

**DOI:** 10.1016/j.ijscr.2025.111904

**Published:** 2025-09-05

**Authors:** Ahmad Alkheder, Adel Azar, Yasser Al-Ghabra, Khaled Almooh, Aliaa Alaitouni, Ahmad Mustafa

**Affiliations:** a Department of Otorhinolaryngology, Al Mouwasat University Hospital, Damascus University, Damascus, Syria; b Faculty of Medicine, Damascus University, Damascus, Syria; c Pathology Department, Al Mouwasat University Hospital, Damascus University, Damascus, Syria

**Keywords:** Antrochoanal polyp, Orbital extension, Coagulopathy, Angiomatous polyp, Endoscopic sinus surgery

## Abstract

**Introduction:**

Antrochoanal polyps (ACPs) typically extend posteriorly into the choana and nasopharynx; orbital invasion is exceptionally rare. This report details an atypical ACP with orbital extension in a coagulopathic patient, highlighting diagnostic and surgical complexities.

**Case presentation:**

A 46-year-old woman with severe Factor V deficiency (0.8 %) presented with 2 years of progressive left nasal obstruction, rhinorrhea, headaches, and snoring. Examination revealed a left nasal polyp extending to the vestibule and bilateral turbinate hypertrophy. Coagulation profiles showed marked prolongation (PTT 126.8 s, INR 3.45). CT imaging identified a hypodense polyp originating from the left maxillary sinus, expanding through the infundibulum into the choana. Crucially, MRI confirmed orbital fossa invasion through bony dehiscence, with T2 hyperintensity and no gadolinium enhancement excluding malignancy. Histopathology post-functional endoscopic sinus surgery (FESS) demonstrated an inflammatory, angiomatous polyp featuring telangiectatic vasculature and stromal hemorrhage.

**Discussion:**

Orbital extension likely resulted from chronic erosion of the lamina papyracea, exacerbated by mass effect. Angiomatous histology—uncommon in adults—and profound coagulopathy amplified bleeding risks. Multidisciplinary management (hematology/ENT) guided preoperative factor replacement and hypotensive anesthesia. Angled endoscopes facilitated precise dissection at the orbital interface, avoiding combined approaches (e.g., Caldwell-Luc) due to coagulopathy. This case underscores MRI's indispensability in delineating atypical extensions and the need for tailored techniques to ensure complete resection amid coagulopathies.

**Conclusion:**

This first reported orbital invasion by an ACP in a Factor V-deficient patient illustrates that benign polyps may erode critical boundaries under chronic pressure. Vigilance for aberrant extensions via advanced imaging, coupled with individualized surgical planning for coagulopathic patients, is essential to mitigate recurrence and complications.

## Highlights

• First orbital invasion by an antrochoanal polyp documented in a Factor V-deficient patient (0.8%), showcasing extreme anatomical deviation under chronic pressure.
• MRI proved critical for detecting subtle orbital fossa extension via bony dehiscence, differentiating this benign polyp from malignancy when CT showed no overt destruction.
• Severe coagulopathy demanded tailored surgery, multidisciplinary planning guided factor replacement and endoscopic-only resection, avoiding combined approaches to minimize bleeding risk.
• Angiomatous histology with fragile telangiectatic vessels in this middle-aged adult amplified hemorrhage risk, a rare finding typically seen in pediatric cases.
• This striking case underscores that benign polyps can erode critical barriers, necessitating advanced imaging vigilance and individualized surgical strategies for complex extensions in high-risk patients.

## 1 Introduction

Antrochoanal polyps (ACPs) are benign, solitary lesions arising from the mucosa of the maxillary sinus, extending through the sinus ostium into the nasal cavity and often protruding posteriorly into the choana and nasopharynx [1,2]. These polyps account for approximately 4–6 % of all nasal polyps and are more commonly observed in children and young adults, though they can present across a broader age spectrum [1,2]. Clinically, ACPs typically manifest with unilateral nasal obstruction, rhinorrhea, and occasionally symptoms such as headaches or snoring, reflecting their obstructive nature [1]. Diagnosis is established through a combination of nasal endoscopy and imaging modalities, including computed tomography (CT) and magnetic resonance imaging (MRI), which delineate the extent and origin of the polyp [1,3].

While ACPs generally follow a predictable anatomical course confined to the nasal cavity and nasopharynx, extension into adjacent structures is rare and represents an atypical presentation [1]. This case report describes a woman with an antrochoanal polyp that uniquely extended into the orbital fossa. This unusual anatomical involvement introduced significant diagnostic and therapeutic challenges. This case emphasizes the importance of recognizing rare extensions of ACPs.

## 2 Methods

This work is also reported in line with SCARE criteria which helped to improve the transparency and quality of this case report [4].

## 3 Case presentation

**Fig. 1:**
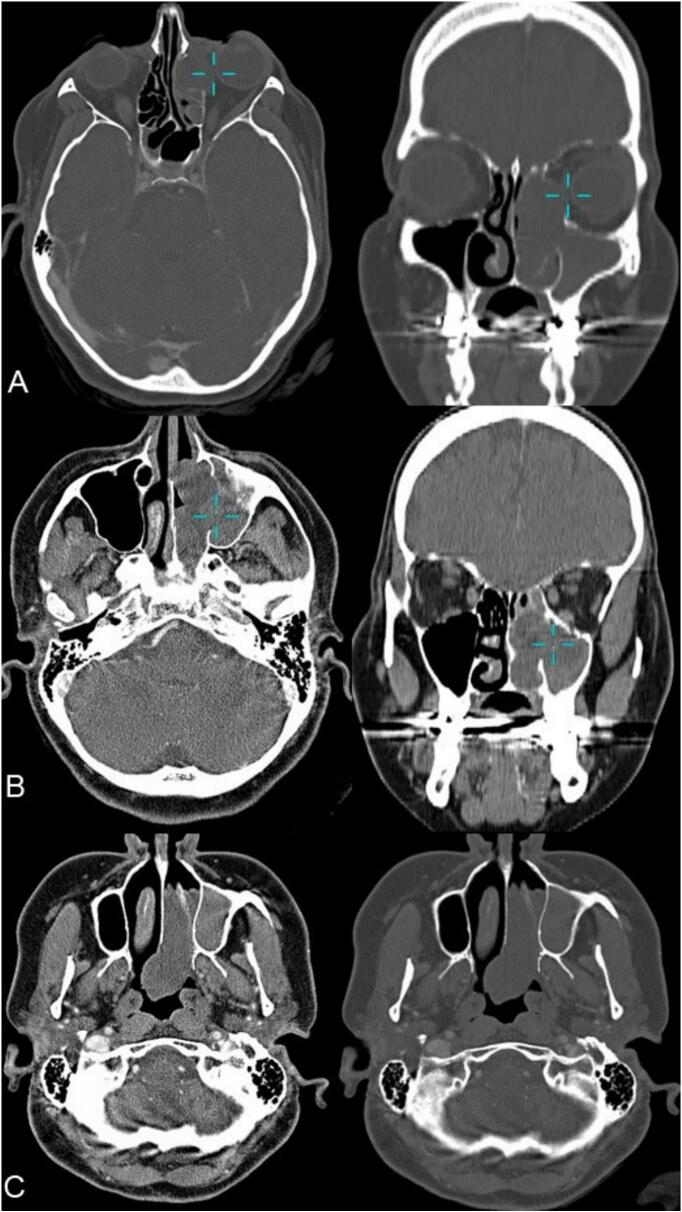
CT scans showing A: a bone window demonstrating a lesion impinging on the orbit. B: a tissue window highlighting a maxillary antrochoanal polyp enlarging the maxillary ostium. C: bone and tissue windows illustrating the extension into the choana.

**Fig. 2:**
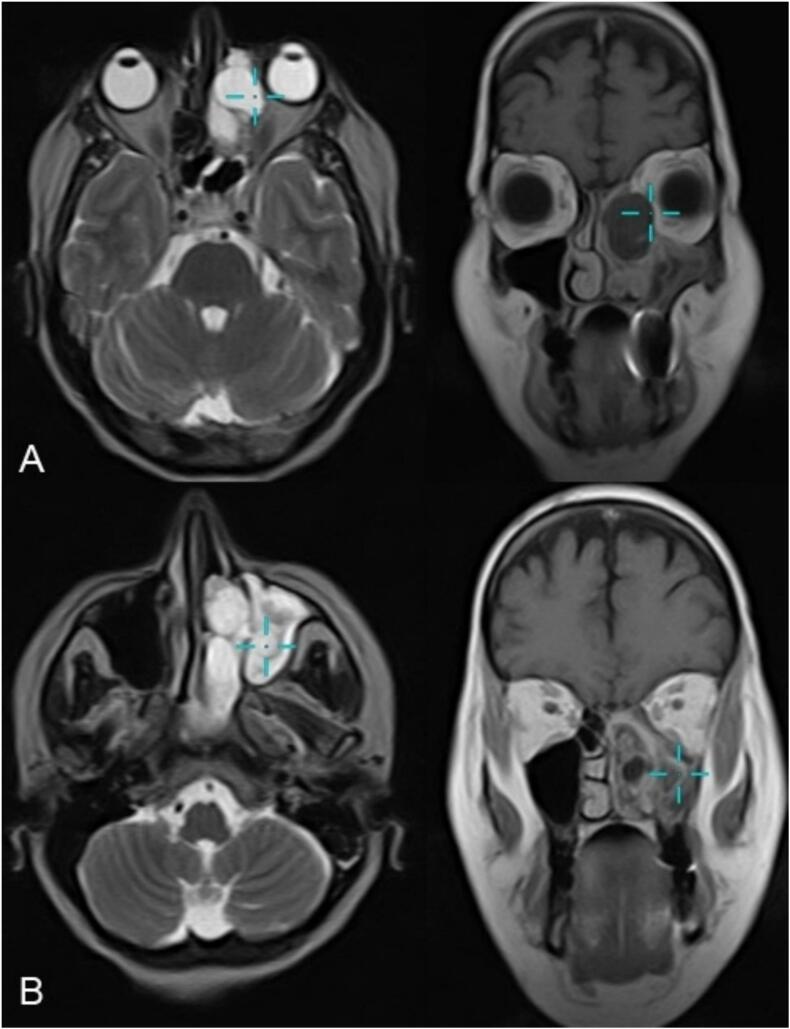
MRI images. A: Axial T2-weighted image showing a bright lesion impinging on the orbit. The corresponding coronal section with T1-weighted imaging post‑gadolinium contrast reveals a lesion that does not enhance. B: The lesion located within the maxillary sinus, also showing no uptake of gadolinium enhancement.

**Fig. 3:**
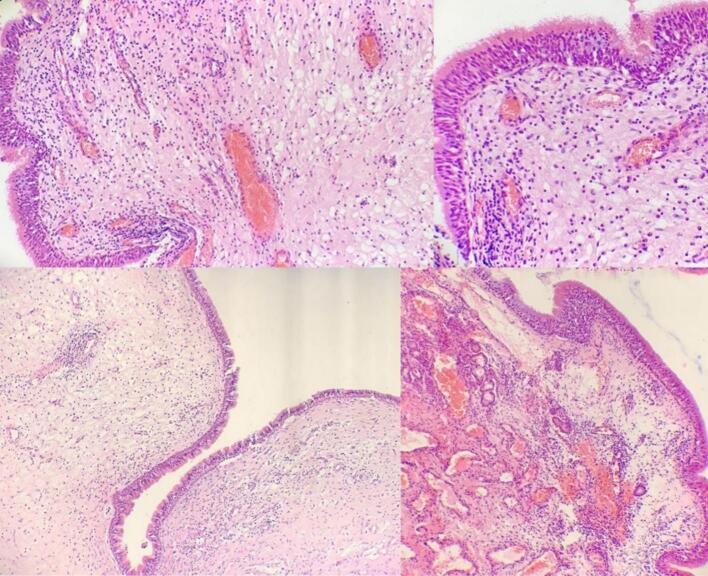
An inflammatory polyp with edematous, loosely myxoid stroma covered by benign respiratory epithelium, infiltrated by mixed inflammatory cells, including lymphocytes, plasma cells, neutrophils, and eosinophils, and exhibiting telangiectatic vasculature and hemorrhage within the stroma.

A 46-year-old woman presented to the ENT clinic with a two-year history of progressive left-sided nasal obstruction, accompanied by occasional clear rhinorrhea, left-sided headaches, mouth breathing, and snoring, despite intermittent use of steroid nasal spray and oral Prednisone for underlying nasal allergies. Notably, the patient reported no preoperative orbital symptoms. Her medical history included Factor V deficiency and hypertension. Physical examination revealed a red polyp emerging from the left middle meatus and extending to the nasal vestibule, along with bilateral inferior turbinate hypertrophy. Laboratory investigations demonstrated severe Factor V deficiency (0.8 %), blood type O+, and markedly prolonged coagulation parameters (PTT 126.8 s, INR 3.45, PT 46 s), though Factor X levels were normal. A CT scan identified a hypodense choanal polyp originating from the left maxillary sinus, widening the infundibulum and extending into the middle and superior meatus, with posterior expansion into the left choana and nasopharynx [Fig. 1]. MRI conducted with T2-weighted and gadolinium-enhanced T1-weighted sequences offered additional insights into the nature of the lesion (Fig. 2). Given the refractory symptoms and imaging findings, she underwent functional endoscopic sinus surgery (FESS) for polyp excision, with preoperative consideration of her coagulation abnormalities. Histopathological examination revealed an inflammatory polyp with edematous, loosely myxoid stroma containing telangiectatic vasculature and hemorrhage within the stroma (Fig. 3).

## 4 Discussion

This report presents an exceptional case of an antrochoanal polyp (ACP) with orbital fossa invasion—a rare deviation from the classical posterior trajectory toward the choana and nasopharynx [1,5,6]. While ACPs typically arise from the maxillary sinus mucosa and expand into the nasal cavity and nasopharynx [1,5,7], orbital extension is scarcely documented. This 46-year-old woman's case underscores the potential for ACPs to erode bony boundaries under chronic inflammatory pressure, despite their benign histopathology [[1], [2], [3]].

Orbital invasion likely resulted from erosion or congenital dehiscence of the paper-thin lamina papyracea, exacerbated by chronic obstruction and mass effect [3,7]. CT findings of infundibular widening and involvement of the middle/superior meatus supported this mechanism [1,3]. Frosini et al. hypothesize that increased intrasinal pressure from ostial obstruction may force mucosal herniation through accessory pathways [7]; here, compromised anatomical barriers permitted anterior-superior extension into the orbit. Such growth risks complications like proptosis or diplopia if untreated [1,7].

MRI (T2 hyperintensity, heterogeneous gadolinium enhancement) was pivotal in delineating orbital involvement and differentiating the ACP from malignancies (e.g., inverted papilloma) or vascular tumors (e.g., angiofibromas) [3,6,8]. While CT confirmed a hypodense choanal polyp without overt bone destruction, subtle bony dehiscence enabled orbital invasion [3,8]. This underscores MRI's indispensability for evaluating atypical extensions near critical structures [8].

Histology revealed an edematous stroma with telangiectatic vasculature and hemorrhage, aligning with the “angiomatous” subtype [6]. Though more prevalent in pediatric ACPs (18 % vs. 5.3 % in adults) [6], this middle-aged presentation suggests chronic mechanical stress or intrinsic vascular fragility. Angiomatous subtypes correlate with higher recurrence due to stromal fragility and rich vascularity [6], compounded here by severe Factor V deficiency (0.8 %). Complete resection is critical, as recurrence rates reach 8–14 % in adults and 18.4 % with standard endoscopic techniques [1,8].

Preoperative steroids provided minimal relief, consistent with ACPs' poor response to medical management [1,3]. Surgery remains definitive. Functional endoscopic sinus surgery (FESS) addressed nasal and antral components. For complex orbital extensions, adjunct approaches (e.g., mini-Caldwell Luc) may optimize access to anterior/inferior maxillary walls [1,3,8], though coagulopathy precluded combined techniques here. Angled endoscopes and microdebriders enabled precise dissection at the orbital interface [7]. Multidisciplinary planning (hematology/ENT) mitigated bleeding risks via factor replacement, hypotensive anesthesia, and staged resection (Single surgery) [2,3,8]. Meticulous hemostasis was imperative to prevent orbital hematoma.

Postoperatively, the patient had an uncomplicated recovery with no orbital sequelae and remained free of recurrence at the 6 -month follow-up.

This case highlights three key deviations from classical ACP behavior: 1) Orbital invasion necessitates vigilance in imaging to detect bony erosion/dehiscence [3,8]. 2) Adult angiomatous histology may signal heightened bleeding propensity, especially with coagulopathies [1,6]. 3) Complex extensions demand tailored surgical approaches to ensure complete resection and reduce recurrence [1,3,8].

Bilateral turbinate hypertrophy here may have altered sinonasal pressure dynamics, promoting polyp initiation [7]. Postoperative intranasal corticosteroids were prioritized to control residual inflammation [6]. Long-term endoscopic surveillance is essential given the polyp's high-risk features [1,8].

## 5 Conclusion

This exceptional case underscores that antrochoanal polyps (ACPs), though typically benign and confined to the sinonasal tract, can exhibit rare and clinically significant extensions, such as orbital invasion. The presentation in a middle-aged adult with angiomatous histology and severe Factor V deficiency further deviates from the classical profile, amplifying diagnostic and surgical complexity. It highlights the critical role of MRI in delineating atypical involvement near vital structures and the necessity for tailored surgical planning, particularly when managing coagulopathies, to ensure complete resection and mitigate recurrence risks. Vigilance for such unusual extensions during evaluation of refractory sinonasal obstruction is paramount.

## Ethical approval

Ethics clearance was not necessary since the University waives ethics approval for publication of case reports involving no patients' images, and the case report is not containing any personal information. The ethical approval is obligatory for research that involve human or animal experiments.

## Guarantor

The corresponding author takes the full responsibility of the work.

## Provenance and peer review

Not commissioned, externally peer-reviewed.

## Registration of research studies

This case report is not a first time of reporting, new device or surgical technique. So I would not need a Research Registry Unique identifying number (UIN).

## Sources of funding

This research did not receive any specific grant from funding agencies in the public, commercial, or not-for-profit sectors.

## Funding

N/A. We received no funding in any form.

## Consent of patient

Written informed consent was obtained from the patient for publication of this case report and accompanying images. A copy of the written consent is available for review by the Editor-in-Chief of this journal on request.

## Declaration of competing interest

The Authors disclose no conflicts.

## Data Availability

All data are available from the corresponding author on reasonable request. The case has not been presented at a conference or regional meeting.
